# Synthesis of Biomass Polycarboxylate Superplasticizer and Its Performance on Cement-Based Materials

**DOI:** 10.3390/ma18143416

**Published:** 2025-07-21

**Authors:** Zefeng Kou, Kaijian Huang, Muhua Chen, Hongyan Chu, Linye Zhou, Tianqi Yin

**Affiliations:** 1 College of Civil Engineering, Nanjing Forestry University, Nanjing 210037, China; nl_zfk95@163.com (Z.K.); chuhongyan@njfu.edu.cn (H.C.); tim1061910871@163.com (T.Y.); 2 College of Chemical Engineering, Nanjing Forestry University, Nanjing 210037, China; simonzrh@163.com (M.C.); 18661158576@139.com (L.Z.)

**Keywords:** cellulose, polycarboxylate superplasticizer, performance, biomass materials

## Abstract

Polycarboxylate superplasticizer (PCE) is an important part of improving the overall performance of concrete. However, its synthetic raw materials are overly dependent on petrochemical products, and it also causes problems such as environmental pollution. With the development of the building material industry, the demand for petrochemical resources required for synthetic water-reducing agents will increase rapidly. Therefore, there is an urgent need to transition the synthetic raw materials of PCE from petrochemicals to biomass materials to reduce the consumption of nonrenewable resources as well as the burden on the environment. Biomass materials are inexpensive, readily available and renewable. Utilizing biomass resources to develop good-performing water-reducing agents can reduce the consumption of fossil resources. This is conducive to carbon emission reduction in the concrete material industry. In addition, it promotes the high-value utilization of biomass resources. Therefore, in this study, a biomass polyether monomer, acryloyl hydroxyethyl cellulose (AHEC), was synthesized from cellulose via the reaction route of ethylene oxide (EO) etherification and acrylic acid (AA) esterification. Biomass polycarboxylate superplasticizers (PCE-Cs) were synthesized through free radical polymerization by substituting AHEC for a portion of the frequently utilized polyether monomer isopentenyl polyoxyethylene ether (TPEG). This study primarily focused on the properties of PCE-Cs in relation to cement. The findings of this study indicated that the synthesized PCE-C5 at a dosing of 0.4% (expressed as mass fraction of cement) when the AHEC substitution ratio was 5% achieved good water reduction properties and significant delays. With the same fluidity, PCE-C5 could enhance the mechanical strength of cement mortar by 30% to 40%. This study utilized green and low-carbon biomass resources to develop synthetic raw materials for water-reducing agents, which exhibited effective water-reducing performance and enhanced the utilization rate of biomass resources, demonstrating significant application value.

## 1. Introduction

Concrete mainly consists of cementitious materials, aggregates, water, admixtures, and supplements. Water is mainly involved in the hydration reaction of cement. If agglomeration occurs in cement, it reduces the degree to which the cement hydration reaction occurs and makes the distribution of hydration products uneven. This reduces the workability and mechanical properties of the resulting concrete. As the “fifth component” of modern concrete technology, concrete admixtures can markedly enhance the fluidity, strength, and durability of concrete [1,2,3]. Water-reducing agents, as significant admixtures, primarily serve to decrease the initial water content in the cement paste so as to enhance its fluidity. At the same time, the decrease in water usage leads to a reduction in the porosity of the hardened material and an increase in its mechanical strength, thus extending the service life of the resulting concrete. Polycarboxylate superplasticizer (PCE) was invented in 1981 [4]. PCE consists of a polyelectrolyte main chain (with anionic groups) and a nonionic polyethylene glycol (PEG) side chain [5,6,7]. The DLVO theory points out that the stability of the colloidal system is determined by the van der Waals force and double-layer repulsion. When the surface charge of the colloidal particles changes, a double-layer structure forms around them. When two charged particles are close to each other, the double layer produces electrostatic repulsion. Thus, particle aggregation and sinking can be inhibited [8]. PCE is adsorbed on the surface of cement through the adsorption groups on the main chain, which improves electrostatic repulsion. At the same time, the steric hindrance provided by the side-chain stretching to the solution breaks the flocculating structure of cement, which improves the dispersibility of cementitious materials using PCE. Therefore, the resulting material has a high water reduction rate and a long duration of flow retention [9,10,11,12]. Because of the strong designability of the molecular structure of PCE, nowadays, in addition to the water-reducing type, PCEs have been developed with a variety of functions to meet the requirements of various construction conditions, including slump retention, early strength, and muck resistance. Winnefeld et al. [13] concluded that as the length of a PCE’s side chain increases, the more prominent its early strength property is. Ran et al. [14] concluded that the length of the side chains of PCE has an effect on slump retention. Long side chains have better dispersing performance than short side chains, and a reasonable length of the main side chain can balance dispersing performance and slump-preserving performance. However, polycarboxylic acid water-reducing agents are costly and overly dependent on petrochemicals for synthetic raw materials [15]. Therefore, in recent years, in order to execute the principles of low carbon and environmental conservation, the advancement and application of green building materials have become a research focus [16,17]. Water-reducing agents with good performance can be prepared through a series of chemical modifications based on biomass materials. This process can significantly decrease production costs and environmental pollution. Consequently, it holds substantial importance for the advancement of sustainability.

As the most widely distributed natural polymer material in nature, cellulose is widely present in all kinds of living organisms in the biosphere. The whole plant system (including cotton and hemp crops, woods, agricultural waste straw, bagasse, etc.) and marine biological resources are especially rich in cellulose resources [18]. A major resource of cellulose is wood. Cellulose has the characteristics of low pollution, extensive distribution, ample reserves, low cost, renewability, and ease of degradation; therefore, it has broad application prospects. Cellulose is a linear polymer chain formed by β-1,4-glycosidic bonds with glucopyranose as the basic structural unit [19]. Its molecular structure contains an extensive quantity of reactive hydroxyl groups. Based on the chemical reactivity of hydroxyl groups, cellulose can participate in a variety of derivatization reactions, such as oxidation, esterification, etherification, cross-linking, and graft copolymerization [20], thus broadening its functions and applications.

Based on current research advances, it is very promising to synthesize water-reducing agents by changing the physicochemical properties of cellulose through various modification techniques. Cheng et al. [21] modified cellulose through hydrochloric acid hydrolysis and grafting maleic anhydride to obtain a cellulose-based polycarboxylic acid water-reducing agent (CBPCS). Carboxylmethyl cellulose sulfate (CMC-S) was synthesized by Huang [22]. It was concluded that CMC-S has a delaying effect. The unsubstituted hydroxyl groups on the CMC-S molecule delayed the hydration reaction of the cement, thereby increasing setting time. As a cellulose derivative, CMC-S has remarkable potential in cement and concrete as an additive with both water-reducing and delaying functions. Maimaiti et al. [23] first hydrolyzed cellulose with acid to obtain microcrystalline cellulose (MCC). Then, it was reacted with 1,4-butanesulfonolactone (BS) and sodium hydroxide to obtain butylsulfonated cellulose ether water-reducing agent (SBC). It was found that SBC is a slow-setting water reducer that can reduce the early hydration rate of cement. Vieira et al. [24] hydrolyzed cellulose to obtain the levelling-off degree of polymerization (LODP) cellulose and, afterwards, chemically modified it to obtain carboxymethyl hydroxyethyl cellulose (CMHEC). It was found that CMHEC has low viscosity and similar dispersing properties for mortar and concrete as naphthalene sulfonic acid formaldehyde condensate (Liquiment N). Paiva et al. [25] investigated the effect of methyl hydroxypropyl cellulose ether (MHPC) on the rheological properties of cement. The dosage of MHPC ranged from 0 to 0.08%. With the increase in MHPC, the plastic viscosity of the cement paste decreased. MHPC showed a high plasticizing effect. Moreover, it delayed the hydration process of the cement particles, which resulted in the prolongation of cement setting time. Bian et al. [26] prepared PC-g-CNCs by grafting cellulose nanocrystals to PCE. PC-g-CNCs effectively improved the mortar fluidity, which resulted in the absence of segregation or water seepage and increased the retention of mortar fluidity. This may be attributed to the high water retention of CNCs, which changed the viscosity of the mortar and also delayed the hydration of the cement, and PC-g-CNCs are expected to be a potential alternative to high-efficiency water reducers. Starch is also a key raw material for biomass energy and biomass materials industries. Zhang et al. [27] prepared a new type of water-reducing agent via esterification modification using corn starch as a raw material. It was found that the long side chains in the structure can produce spatial site resistance to cement. At the same time, the ester group introduced in the molecular structure gives it the property of delaying. Isik et al. [28] used chemically modified starch ether to produce self-compacting concrete. The results showed that it could reduce the viscosity of the concrete mix. The concrete slump was not less than 60 cm and became more resistant to segregation.

Cellulose ethers (CEs) are a class of cellulose derivatives synthesized through the reaction of fundamental cellulose with etherifying agents under specific conditions [29]. Cellulose ether (CE) is a critical additive for a category of building materials that can contribute to the delay, anti-settling, and water retention of cementitious materials [29,30]. CE has the potential to delay the hydration of cement, thereby extending its setting time [31,32]. As one of the cellulose ethers, hydroxyethyl cellulose ether (HEC) contains hydrophilic ether bonds in its structure. Meanwhile, the active hydroxyl groups on the molecular chain of HEC can react with many groups to prepare multifunctional polymer materials [33].

All of the above water-reducing agents are prepared by using cellulose and its derivatives as raw materials and introducing hydrophilic groups through a series of chemical modifications [34]. Although there are many studies on the synthesis of water-reducing agents using cellulose and its derivatives, there is still a gap in the water reduction rate of high-performance PCE. The novelty of this study lies in the synthesis of a novel biomass polyether monomer, AHEC, by introducing polymerizable allyl groups into the cellulose via an innovative chemical modification route of ethylene oxide etherification and acrylate esterification. This monomer can replace part of the traditional petroleum-based polyether macromonomer TPEG as an important raw material for synthesizing PCE. This research aims to develop an environmentally friendly, high-performance, and sustainable PCE product that will reduce the concrete admixture industry’s dependence on fossil resources. Meanwhile, the effects of different substitution ratios of AHEC for TPEG in the reaction components of synthesized PCE on the properties of the gelling materials were investigated to establish the relationship between the microscopic mechanism and the macroscopic properties.

## 2. Materials and Methods

### 2.1. Materials

Cement P.O 42.5 (Conch, Nanjing, China) was used. The chemical composition and technical performance indices of cement are presented in Table 1 and Table 2. The coarse aggregate consists of crushed basalt stone, while the fine aggregate comprises medium sand. The aggregate’s particle size is presented in Table 3 and Table 4. Chemicals used in this study are presented in Table 5.

### 2.2. Methods

In this study, cellulose was first etherified using ethylene oxide (EO) to obtain hydroxyethyl cellulose polyether (HEC) with good water solubility. Then, AHEC and acrylic acid (AA) were esterified to obtain the biomass polyether monomer AHEC with polymerization activity. Finally, the biomass polycarboxylate superplasticizer PCE-Cs was synthesized through free radical polymerization by replacing a part of the commonly used polyether monomer TPEG with AHEC.

#### 2.2.1. Synthesis of AHEC

The polyether monomers in the synthesized raw materials of polycarboxylic acid water-reducing agents are usually terminal alkenyl macromonomers with terminal double bonds. Therefore, to prepare biomass polyether monomers, hydroxyethyl cellulose (HEC) needs to be modified. The core of the modification method is to graft carbon–carbon double bonds to HEC to make it a monomer with polymerization activity, so that it can be polymerized with the monomers of the polycarboxylic acid water-reducing agent in the later stage. The synthesis of AHEC is primarily categorized into two stages: Firstly, cellulose is etherified with ethylene oxide to obtain HEC. Secondly, biomass polyether monomer AHEC is formed through the esterification of HEC and AA.

We weighed the appropriate amount of cellulose, and the concentration of 20% sodium hydroxide solution was reacted at 25 °C. After sufficient stirring and mixing, the reaction is allowed to proceed for 2 h, and the filtrate is obtained after the product is dried in an 80 °C oven for 24 h. Finally, through grinding and sieving, the powder obtained is alkali cellulose. NaOH solution can not only deconstruct the crystalline regions of cellulose and improve the hydroxyl reaction activity but also react with the hydroxyl group of cellulose to generate sodium alcohol, which can significantly improve the nucleophilicity and, thus, improve the efficiency of the subsequent reaction with ethylene oxide [35].

**Figure 1:**
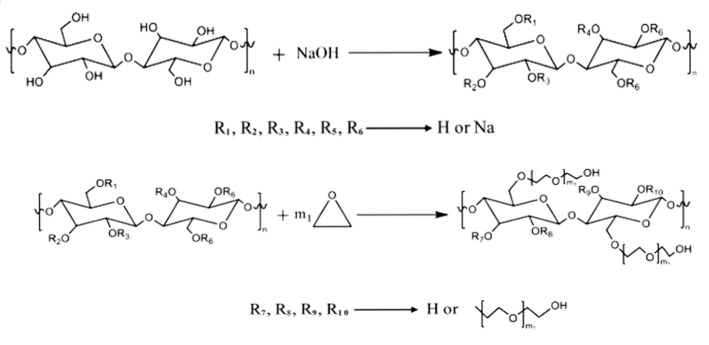
Reaction equation for the synthesis of HEC.

**Figure 2:**
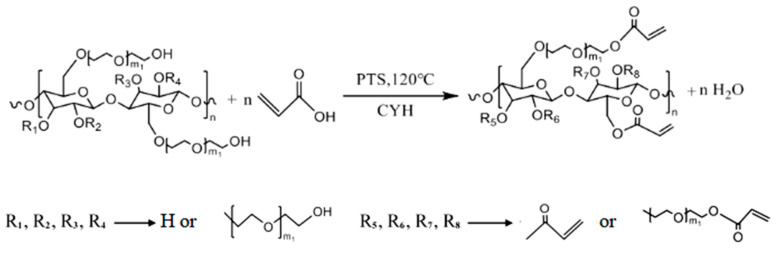
Reaction equation for the synthesis of AHEC.

Alkali cellulose was dissolved in the dispersant xylene and put into the reactor, and ethylene oxide was added in a dropwise feeding mode to control the reaction rate. The air in the reactor should be removed before the test. The mass ratio of alkali cellulose to ethylene oxide was 1:5. We turned on the stirrer, and 90 °C was maintained as the reaction temperature. The reaction ends when the drop of ethylene oxide is finished, until the pressure of the system no longer changes. Then, a diluted hydrochloric acid solution was used to bring the system’s pH down to neutral to terminate the reaction and remove the residual alkali. Finally, the yellowish-brown viscous liquid HEC was obtained after several water washes and spin evaporation. The prepared HEC was dissolved in cyclohexane (CYH), and then acrylic acid and catalyst p-toluenesulfonic acid (PTS) were added; the molar ratio of AA to HEC was 1:2, the reaction temperature was kept at 120 °C, and the reaction time was 7 h. Finally, AHEC was obtained via rotary evaporation. The iodine value was used to characterize the double-bond grafting ratio. The test method is described in Section 2.2.3. Too many or too few grafted double bonds are detrimental to the subsequent polymerization reaction. Based on the iodine value of the replaced TPEG, the ratio of AA was adjusted to control the iodine value of the products. AHEC has an iodine value of 7.7 g/100 g. TPEG has an iodine value of 9.6 g/100 g. The reaction equation is shown in Figure 1 and Figure 2.

#### 2.2.2. Synthesis of PCE-Cs

**Figure 3:**
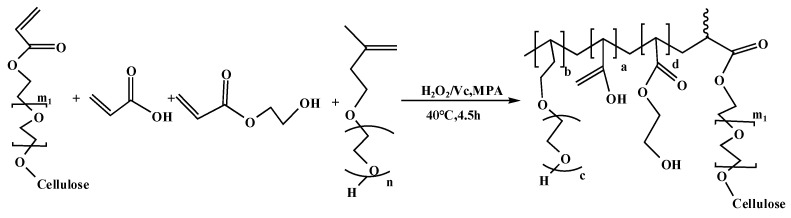
Reaction equation for the synthesis of PCE-Cs.

**Figure 4:**
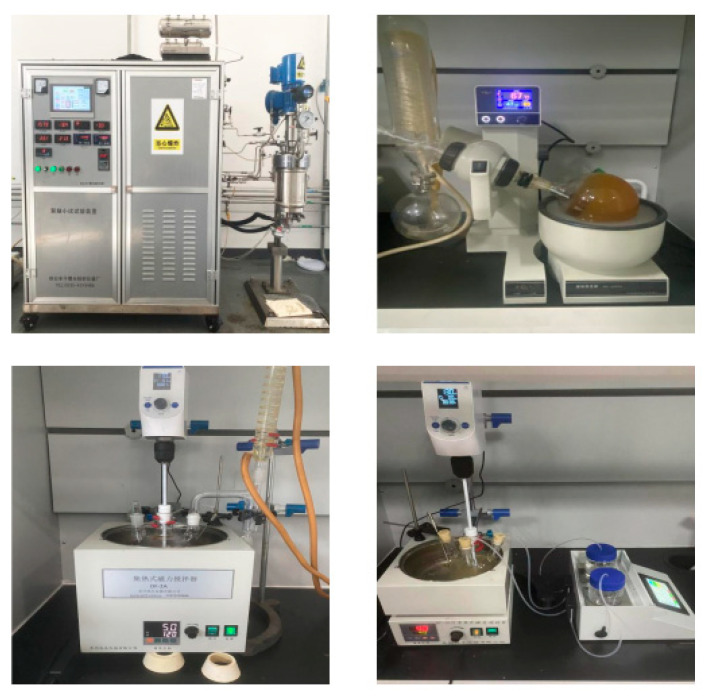
Flowchart for the preparation of AHEC and PCE-Cs.

**Figure 5:**
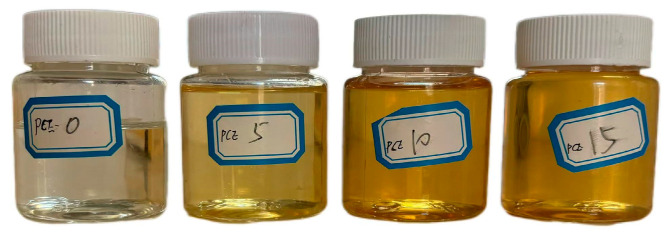
Product diagram of PCE-Cs.

Introduce a specified quantity of AHEC and TPEG into a flask, add deionized water, heat and stir to dissolve the monomer, control the temperature at 40 °C, add 30% hydrogen peroxide (H_2_O_2_) solution, configured ahead of time. Solution A (AA and HEA) and solution B (L-ascorbic acid and mercaptopropionic acid) are put through the peristaltic pump to a certain drop acceleration to add to the four-necked flask. Solution A needs to be controlled for 3 h and solution B for 3.5 h. After the end of dropwise addition, the reaction was then carried out at a constant temperature for 1 h. After the reaction was complete, the pH was adjusted to neutral, and the water-reducing agent PCE-CX was obtained, where X is the mass percentage of AHEC replacing TPEG. Four water-reducing agents, PCE-C0, PCE-C5, PCE-C10, PCE-C15, were synthesized. The reaction equations are shown in Figure 3. Flowchart for the preparation of AHEC and PCE-Cs is shown in Figure 4. Product diagram of PCE-Cs is shown in Figure 5. The masses of raw materials required for synthesizing PCE-Cs are shown in Table 6.

#### 2.2.3. Iodine Value

The iodine value is used to measure the degree of unsaturation of AHEC. The iodine value can reflect the content of grafted double bonds on the structure of the AHEC molecule. The iodine value was determined according to the Chinese standard GB/T 13892-2020 [36]. Weigh the specimen in an iodine measuring flask and dissolve it, add 10 mL of Weiss’ reagent for thorough mixing and leave it for 1 h. Then, add 15 mL of KI solution and 50 mL of water, titrate with sodium thiosulfate solution until the yellow color of iodine is close to disappearing. Finally, add 2 mL of starch solution, continue the titration, and shake vigorously until the blue color disappears. Conduct another set of blank tests according to the above steps.

f = 0.1269c(V_2_ − V_1_)/m (1)

f: iodine value, g/100 g; V_1_: volume of sodium thiosulfate solution consumed for titrating the test material group, mL; V_2_: volume of sodium thiosulfate solution consumed for titrating the blank sample, mL; c: concentration of sodium thiosulfate solution, mol/L; m: mass of test material, g.

#### 2.2.4. Fluidity

The Chinese standard GB/T 8077-2012 [37] was consulted to determine the testing procedure. Weigh 300 g of cement, 87 g of water, and a certain mass of PCE-Cs, and then mix them in a cement paste mixer. A truncated cone circular mold was filled with the mixed cement paste, and the mold was then raised to let the paste run on the glass plate for 30 s. The flowability of cement paste was characterized by measuring the two largest diameters in the vertical direction of the flowed portion with a straight edge and taking the average of the diameters as the flowability of the cement paste. The cement paste’s initial fluidity, as well as its fluidity at 60 and 120 min, was assessed using this approach. Each set of tests was repeated three times.

#### 2.2.5. Water Reduction Rate

Test methods referred to the Chinese standard GB/T 8077-2012 [37]. By adjusting the amount of water required to keep the fluidity of the mastic sand at 180 mm, the water consumption of the mastic sand without PCE-Cs was measured first, the amount of water required to mix PCE-Cs was measured, and then the water reduction rate was obtained after calculation.

#### 2.2.6. Setting Time

The setting time test refers to the Chinese standard GB/T 1346-2011 [38]. Weigh 500 g of cement and 142 g of water and mix to make a standard consistency cement paste. Mix PCE-Cs with the standard consistency cement paste, and then load it into the mold for maintenance and start to calculate the time, with the test pin from the base plate 4 mm ± 1 mm when the cement reaches the initial solidification state and with the test pin sinking into the cement specimen 0.5 mm when the cement reaches the final solidification state. Each set of tests was repeated three times.

#### 2.2.7. Cement Mortar Strength

The Chinese standard (GB/T 17671-2021 [39]) is the basis for the strength test of cement mortar. The PCE-Cs were incorporated into cement mortar, and the fluidity was regulated to 180 mm by modulating water consumption. The resulting cementitious sand was subsequently cured for 24 h in a standard curing environment by placing it in a mold that measured 40 mm × 40 mm × 160 mm. The mold was subsequently removed to allow for further curing. Compressive and flexural strength tests were conducted using an electronic compression and flexural testing machine (electronic compression and flexural testing machine, UTM7305, Shenzhen Suns Test Equipment Co., Ltd., Shenzhen, China) after curing periods of 3, 7, and 28 days. The fine aggregate follows the Chinese ISO standard (GB/T 17671-2021) for sand. The average of the flexural strengths of 3 specimens in each group is taken as the result. When 1 of the 3 strengths exceeds ±10% of the average value, it should be rejected. The average of the compressive strength of 6 specimens in each group is taken as the result. When 1 of the 6 strengths exceeds ±10% of the average value, it should be excluded. When 2 out of 6 strengths exceed ±10% of the average value, this group of tests is invalidated.

#### 2.2.8. GPC

Molecular weight testing of products is performed using an Agilent 1260 Infinity II liquid chromatograph (Agilent, Santa Clara, CA, USA). The chromatograph was equipped with a refractive index detector. The eluent phase was a 0.1 mol/L NaNO_3_ solution at a flow rate of 1 mL/min. The sample concentration was 2.0 mg/mL, the injection volume was 50 μL, and the test temperature was 40 °C.

#### 2.2.9. FTIR

The samples were measured using a Thermo Nicolet iS5 Fourier infrared absorption spectrometer (Thermo, Madison, WI, USA). The KBr and PCE-Cs samples were dried and treated first, and then the appropriate amount of potassium bromide was ground together with the PC-Cs samples and pressed into a transparent sheet. The testing of PCE-Cs was conducted from 400 cm^−1^ to 4000 cm^−1^.

#### 2.2.10. ζ-Potential

The ζ-potential of PCE-Cs was detected via the electrophoresis method. Test method: Different PCE-Cs aqueous solutions with a concentration of 1 g/100 mL were prepared; 1 g of cement was added to the sample solution, stirred for 5 min, and then left to stand for 1 min; and the upper layer of the clear solution was taken and tested with a Zetasizer Nano ZS90 dynamic light scattering meter (Malvern Panalytical, Worcestershire, UK).

#### 2.2.11. X-Ray Diffraction (XRD)

The crushed specimens were placed in anhydrous ethanol for 1 day to prevent the cement from hydrating, and the cement specimens were cured for 3 days and 7 days. The samples were subsequently pulverized and desiccated to produce a solid powder. Ultima IV X-ray diffraction (Rigaku, Tokyo, Japan) was employed to characterize the composition of cement hydration products. The operating voltage was 60 kV, the power was 2.2 kW, and the scanning range was 10° to 80°. The scanning velocity was 1° per minute.

#### 2.2.12. Thickness of Adsorption Layer

Thus, 1 g of benchmark cement was added to 100 milliliters of PCEs-Cs solution at a concentration of 1 g per liter, stirred for 1 h, and then filtered and separated to obtain the filter cake, which was dried in an oven at 80 °C, ground into powder, and used for XPS analysis. Since there is no Si element in the water-reducing agent, Si can be used as a characteristic element to determine the intensity change of Si2p photoelectrons passing through the adsorbed layer, to calculate thickness of the adsorbed layer. The calculation method can be found in reference [40].

## 3. Results and Discussion

### 3.1. Fluidity of Cement Paste

**Figure 6:**
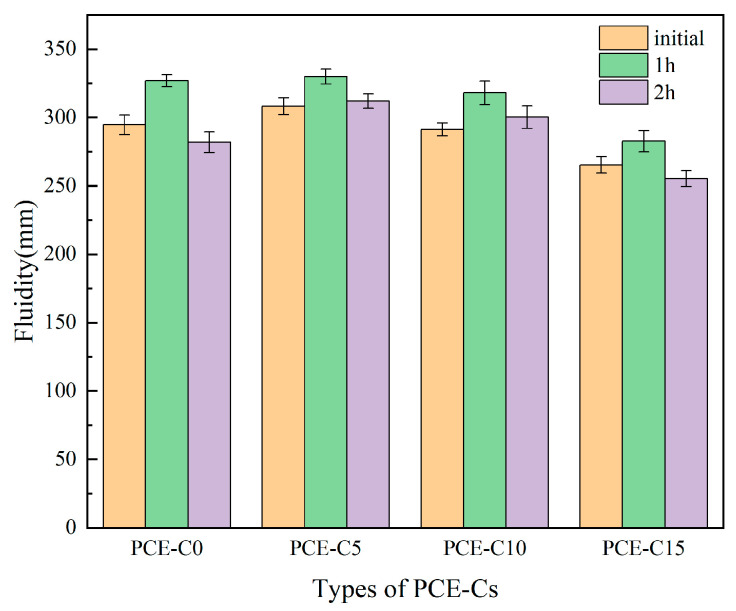
The fluidity of different PCE-Cs. Error bars represent standard deviation (SD) (*n* = 3).

PCE-C0, PCE-C5, PCE-C10, and PCE-C15 were incorporated into the cement paste at a mass fraction of 0.4%. Experiments were performed to assess the fluidity of the cement paste after the start, 1.0 h, and 2.0 h as a means to analyze dispersion and dispersion retention of PCE-Cs on cement. Figure 6 displays the test findings.

Figure 6 illustrates that the initial flowability of PCE-Cs in cement paste diminished as the AHEC substitution ratio increased, resulting in a notable reduction in cement paste flowability from 308 mm to 265 mm when the AHEC substitution ratio rose from 5% to 15%. PCE is adsorbed on cement through carboxyl groups on the molecular chain. The carboxyl group complexes with Ca^2+^. Cement shows electrostatic repulsion due to homogeneous charges. In addition, the steric hindrance created by the side chains of the PCE promotes the dispersion of the cement. As a result, the cement obtains good fluidity. If the side chain of PCE is too short, the steric hindrance is small, and the dispersion performance is not good. If the side chain of PCE is too long, the side chain adsorbed on the surface of cement will be more likely to undergo obvious curling [41]. And the side chain may be combined with part of the free water through hydrogen bonding. So, the freedom of motion of the side chain is restricted. This would reduce the steric hindrance of the side chain. In addition, it may also shield the carboxylate group anchoring point on the main chain of the PCE, which affects the ionization of the carboxylate group and the adsorption of the cement particles. These conditions will reduce the dispersing ability of the cement. The addition of too much AHEC will more easily make the side chain of PCE-Cs entangled and bent, affecting the ionization of carboxyl groups. In addition, the side chain cannot be well stretched, and the steric hindrance will also be weakened, thus affecting the dispersion of cement. Consequently, the fluidity of the cement paste of PCE-C15 will be decreased. The fluidity of PCE-C5 and PCE-C10 reached 308 mm and 291 mm, respectively. The dispersing performance of PCE-C5 is better than that of PCE-C0, which may be attributed to the hydrophilic hydroxyl groups in the cellulose structure. The hydrogen bonding between hydroxyl groups and water molecules results in the formation of a large amount of solvated water film on the surface of cement. The solvated water film can act as a lubricant between the cement, thus enhancing the workability of the cement [42]. In terms of fluidity, PCE-C5 has higher flowability over time compared to Cheng’s test [21]. When the dosage was 0.4%, the flowability after 1 h increased by 7.1%, which had good flowability retention. This is because ester groups of PCE-Cs are gradually hydrolyzed to carboxyl groups in the alkaline environment of cement paste. The carboxyl groups in the liquid phase continue to increase, and the PCE-Cs are continuously adsorbed on the surface of the newly generated hydration products. Therefore, it shows the phenomenon of flowability increasing with time.

### 3.2. Water Reduction Rate of Cement Mortar

Taking the water consumption of cement mortar without PCE-Cs as a benchmark, the water consumption of each water-reducing agent was measured at dosages of 0.2%, 0.4%, 0.6%, and 0.8%, and then the water reduction rate at each dosage of PCE-Cs was calculated.

**Figure 7:**
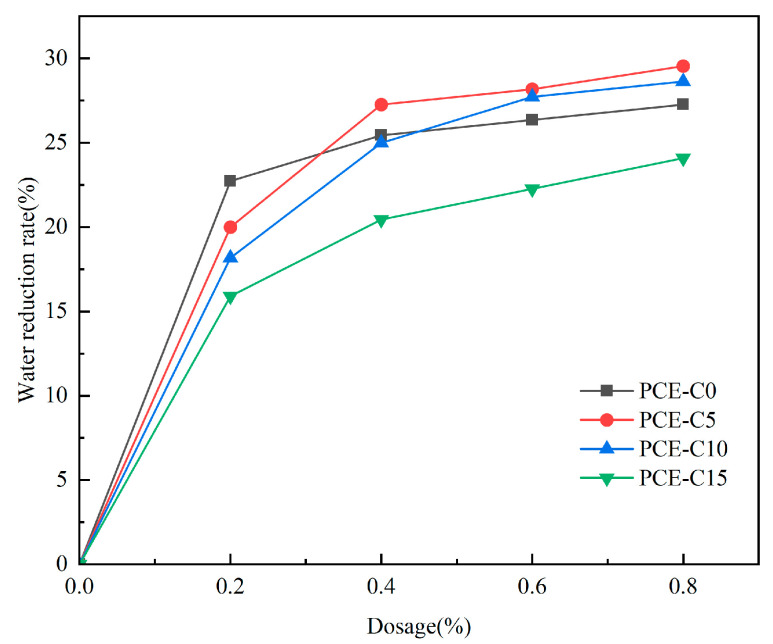
Water reduction rate of different PCE-Cs.

Figure 7 illustrates that the water reduction rate of PCE-Cs progressively increases with the dosage until it stabilizes. At a dosage of 0.2%, the water reduction rate markedly increases with higher dosages, while at 0.4%, the increase in the water reduction rate is minimal. When the dosage is 0.4%, the water reduction in all four water-reducing agents is higher than 20%. These results align closely with the flowability test outcomes. Compared with other water-reducing agents, PCE-C5 has a higher water reduction rate and better dispersing performance in cement. In terms of water reduction, CBPCS prepared by Cheng reached 28.3% at 0.5% dosing [21]. PCE-C5 demonstrates equally high water reduction with CBPCS.

### 3.3. Setting Time of Cement

**Figure 8:**
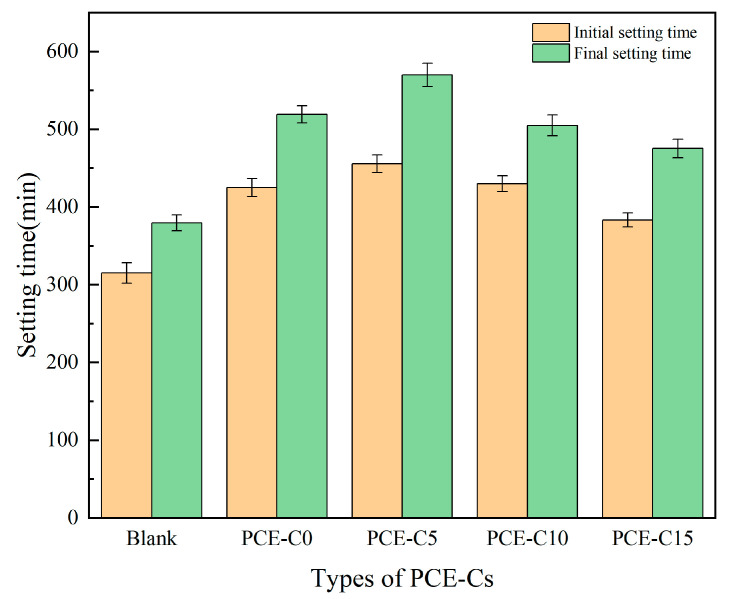
Setting time of different PCE-Cs. Error bars represent standard deviation (SD) (*n* = 3).

As illustrated in Figure 8, when the water consumption of each group was the same, compared with the blank group, the incorporation of PCE-Cs prolonged the setting time of the cement and had certain delaying properties. Particularly, PCE-C5 has a more significant effect on the setting time of the cement paste. PCE-C5 increases the initial setting time by 141 min and the final setting time by 190 min. This is due to the fact that under alkaline cement paste system conditions, the hydroxyl groups on the cellulose structure form unstable complexes with free Ca^2+^. This reduces the concentration of Ca^2+^ and, thus, inhibits the hydration of the cement. In addition, the hydroxyl group in the cellulose structure adsorbed on the surface of cement will also form a hydrogen bond with negative oxygen ions on the surface of hydration products. And other hydroxyl groups can be bonded with water molecules through hydrogen bonding to make the surface of cement form a layer of stable solvated water film, thus inhibiting the hydration process of cement [43,44]. From Figure 8, we can see that the effect of PCE-C0 is not as big as that of PCE-C5. It is mainly because the water-reducing and dispersing effect of PCE-C0 is slightly lower than that of PCE-C5. The fluidity of cement paste mixed with PCE-C5 is greater than that of cement paste mixed with PCE-C0, which releases more free water. Therefore, the time to reach the initial setting state and the final setting state is much longer, so PCE-C5 has a more significant effect on the extension of the setting time of cement paste than PCE-C0.

### 3.4. Mechanical Properties

Water-reducing agents can decrease the water requirement of cement paste, which in turn improves fluidity and significantly increases the cement strength. Additionally, they reduce the water–cement ratio. Consequently, the impact of PCE-Cs on the compressive and flexural strength of cementitious sand was examined. The results of the test are presented in Table 7.

From Table 7, it can be seen that under the condition of the same flow degree, the compressive strength and flexural strength of each group of water-reducing agents have been greatly improved. Among them, PCE-C5 has the most excellent mechanical properties, the 3d compressive strength and 3d flexural strength increased by 49.2% and 41.3% compared with the blank group, and the 7d compressive strength and 7d flexural strength increased by 35.8% and 33.3% compared with the blank group. The 28d compressive strength of 45.1 MPa and 28d flexural strength of 7.4 MPa in the blank group were improved by 32.6% and 22%, respectively. The decrease in water consumption and the significant improvement in the strength of cement mortar with PCE-C5 can be attributed to the excellent dispersing performance of PCE-C5. On the one hand, the hydrophilic hydroxyl and ether groups in the molecular structure of cellulose can improve the water retention of mortar, promote the aggregate and cementitious materials to be fully mixed and homogeneous, and improve the working performance of cement mortar. This makes the mortar less prone to the occurrence of phase separation, so that it is easier to form and develop the mechanical strength [45]. In addition, the improvement in mortar’s working performance also helps to enhance the homogeneity of the overall structure of hardened cement mortar, thus improving the mechanical properties. On the other hand, due to the electrostatic repulsion and steric hindrance provided by PCE-Cs, it can facilitate the dispersion of cement, impede the agglomeration of cement and the generation of flocculation structure, release more free water, and improve the hydration degree of cement. Therefore, it can reduce the water requirement of cement under the same mobility as the blank group and, thus, reduce the water–cement ratio. In general, a lower water–cement ratio results in increased strength. As PCE-C5 has a higher water reduction rate, and its water–cement ratio is smaller, so it can improve the strength of cement mortar more [2]. In terms of strength, compared to the test by Cheng [21], the strength of the cement mortar was higher in this experiment due to the use of a smaller water–cement ratio.

### 3.5. FTIR for HEC and AHEC

**Figure 9:**
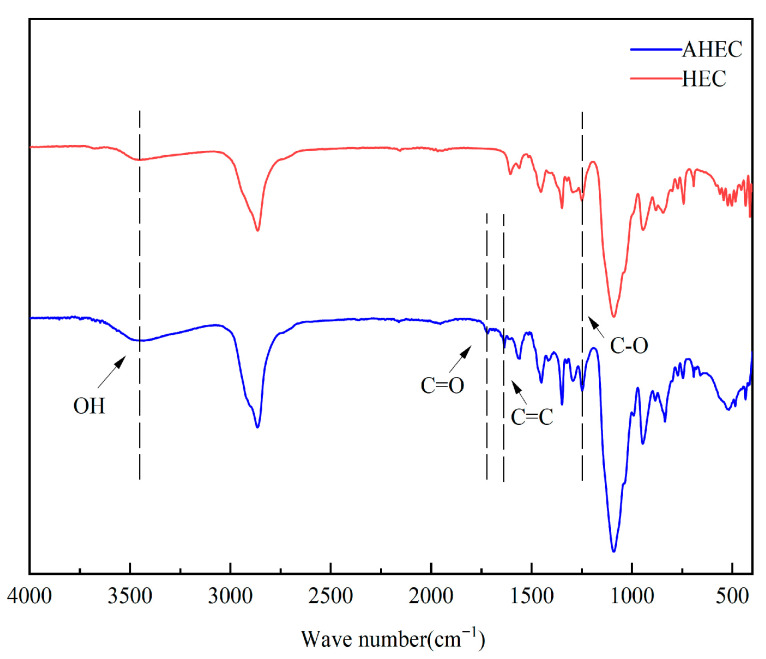
Infrared spectra of HEC and AHEC.

Figure 9 shows the infrared spectra of HEC and AHEC. There is an absorption peak near wave number 3400 cm^−1^, which is the result of the stretching vibration of the alcohol hydroxyl group -OH. The stronger absorption peaks near the wave number 2900 cm^−1^ are the stretching vibration peaks of methylene; the absorption peaks of glycosidic and C-O bonds in the cellulose structure appeared near the wave number 1090 cm^−1^, and these features indicate the existence of the cellulose structure. Meanwhile, a C=C stretching vibration peak can be seen in AHEC near the wave number 1638 cm^−1^, and a carbonyl stretching vibration peak appears near the wave number 1720 cm^−1^, which is a new absorption peak introduced by the esterification reaction between acrylic acid and cellulose ether. In summary, the allyl group was successfully grafted onto HEC.

### 3.6. FTIR for PCE-C0 and PCE-C5

**Figure 10:**
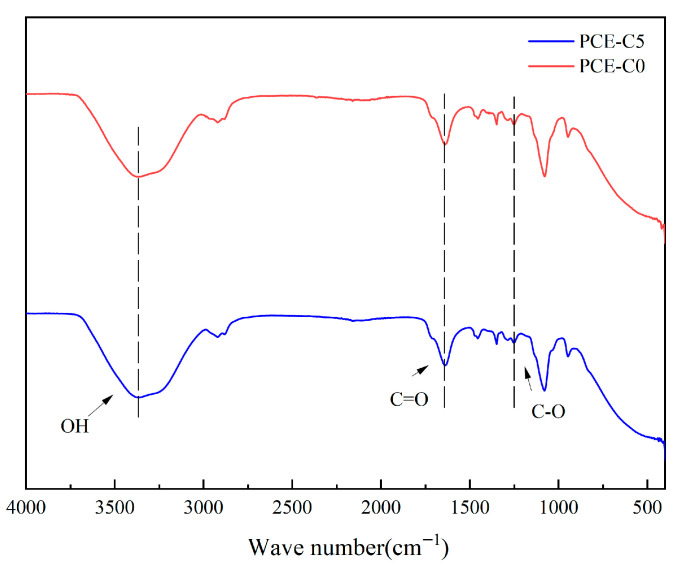
Infrared spectra of PCE-C0 and PCE-C5.

Figure 10 shows the infrared spectra of the water-reducing agents PCE-C0 and PCE-C5. The structural groups of the two water-reducing agents are relatively similar, and the absorption peaks of the samples are not very different, in which the hydroxyl group of the water-reducing agent appears as a telescopic vibration peak in the vicinity of 3357 cm^−1^. The absorption peaks of carboxylic acid carbonyl group C=O appeared in the range of 1650–1750 cm^−1^, 1251 cm^−1^ is the telescopic vibration peak of ether bond C-O-C, and the telescopic vibration near 2900 cm^−1^ corresponds to the telescopic vibration of C-H. These features can indicate that the synthesized polycarboxylic acid water-reducing agent contains carboxylic acid, hydroxyl, ether group, and other groups, and the synthesized water-reducing agent molecular structure conforms to the expected design.

### 3.7. XRD Analysis of Cement Hydration Products

Cement consists mainly of the minerals 3CaO·SiO_2_, 2CaO·SiO_2_, 3CaO·Al_2_O_3_ and 4CaO·Al_2_O_3_·Fe_2_O_3_. Cement begins a complex chemical reaction when water is added to it. The products are mainly composed of CH, AFt, and C-S-H. The effect of PCE-Cs on the hydration process of cement can be analyzed by observing the changes in major hydration products at different ages. And, in this section, the effect of PCE-Cs on the hydration products of cement at 3d and 7d will be analyzed using XRD to further determine whether PCE-Cs can delay the hydration of cement.

**Figure 11:**
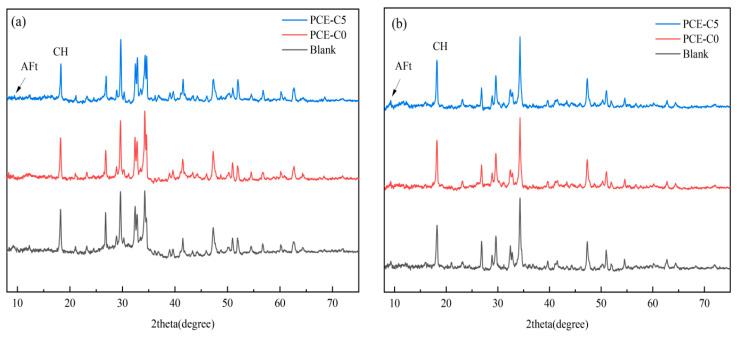
XRD diagrams of cement hydration products at different ages: (**a**) 3d; (**b**) 7d.

Figure 11 show that compared with the hydration products of cement without PCE-Cs, after 3d hydration of cement, the cement doped with PCE-C5 exhibits a weaker characteristic peak of calcium hydroxide CH around 2θ = 18°, indicating that the PCE-C5 doping inhibits the early hydration of the cement and reduces the hydration rate of the cement and, thus, reduces the amount of calcium hydroxide (CH) generated. This is because the hydroxyl group of PCE-Cs forms unstable complexes with free calcium ions in the liquid phase, which reduces their concentration, thereby inhibiting the generation of CH [46]. The small peak at 9.1° with the cement sample is the characteristic peak of AFt. The intensity of the peaks of the cement sample with PCE-Cs is lower than that of the blank group. It can be concluded that the content of AFt is lower. This also indicates that the PCE-C hinders the early hydration of the cement. With the continuous hydration of cement, at 7d, the difference in the intensity of the CH characteristic peaks of the cement specimen samples was not significant, indicating that although PCE-C5 inhibited the early cement hydration and affected the generation of the early hydration products, it did not have a large effect on the later cement hydration reaction.

### 3.8. Gel Permeation Chromatography (GPC)

As can be seen from Table 8, both the number average molecular weight and the weight average molecular weight of PCE-C5 were increased compared to PCE-C0, which was attributed to the introduction of AHEC in PCE-C5. Based on the PDI values, it can be seen that the polymerization products of both PCE-C5 and PCE-C0 are uniformly distributed. According to the monomer conversion rates of the two, it can be seen that both PCE-C5 and PCE-C0 have high monomer conversion rates and a high percentage of target polymerization products, which not only indicates a relatively small percentage of by-products but also shows that the introduced biomass polyether monomers AHEC and other raw materials were successfully polymerized.

### 3.9. Zeta Potentials

**Figure 12:**
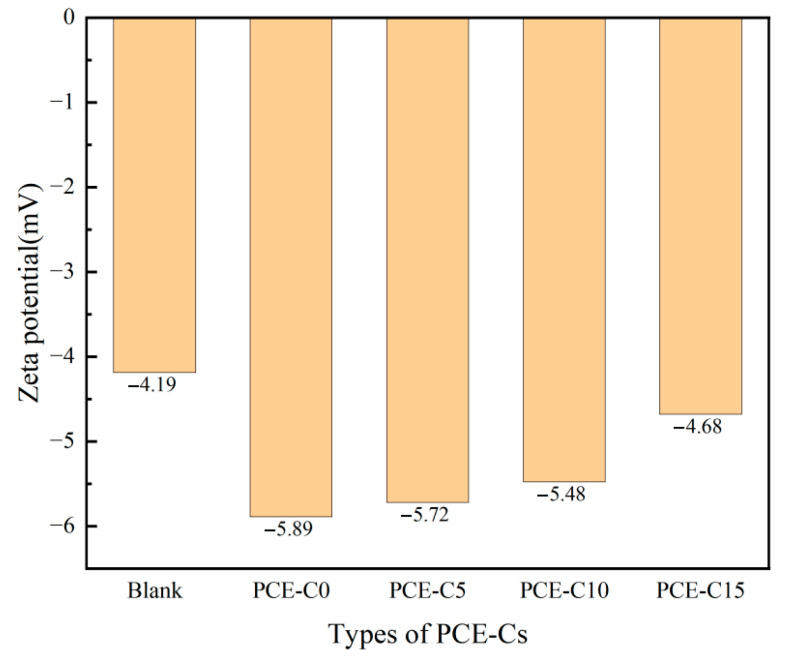
Zeta potential of each water-reducing agent.

To analyze the effect of PCE-Cs on the surface charge of cement particles, the zeta potential of the cement suspensions of PCE-C0, PCE-C5, PCE-C10, and PCE-C15 was tested. Figure 12 displays the test findings. From Figure 12, it is evident that PCE-Cs doping can considerably lower the cement particles’ zeta potential. They are both higher than the blank group.

As for the cement system, the addition of PCE-Cs will change the charge state of cement particles. The anionic groups, such as carboxylate ions in the molecular structure, will combine with the positively charged calcium ions on the cement and form a double layer. As more PCE-Cs are adsorbed on the cement surface, the number of anionic groups (such as carboxylate) increases. This leads to a higher absolute value of the zeta potential. The stronger the electrostatic repulsion between cement particles, the less likely cement agglomeration is to occur. This results in macroscopically improved cement fluidity. Therefore, under general conditions, the absolute value of zeta potential reflects the electrostatic repulsion between cement. From Figure 12, we can also see that the electronegativity of the zeta potential decreases with the increase in the AHEC substitution ratio, and the value of the potential of PCE-C15 is −4.68 mV, which is somewhat lower compared with that of PCE-C0. This is due to the presence of more AHEC in the structure of PCE-C15. This makes it easier for the molecular chain to entangle and agglomerate, resulting in the carboxyl group being easily wrapped by the side chain. This would affect the ionization of the carboxyl group and shield a portion of the negative charge. Consequently, the absolute value of the zeta potential decreased, and the dispersion ability of the cement was also reduced to a certain extent. In addition, when combined with the previous results of the net paste fluidity, it was found that there is a certain difference between the results of the two tests. The dispersibility of PCE-C5 and PCE-C10 for cement paste was better than that of PCE-C0, but there was not much difference in the value of the zeta potential of the three.

Yoshioka et al. [8] determined that a zeta potential of 20 mV is the critical threshold required for the uniform dispersion of cement particles and the stabilization of the system, based on the extrapolation of the repulsive potential energy of cement colloidal bilayers. However, the test results showed that the absolute value of the potential of the cement paste with PCE-Cs was small, so the electrostatic repulsive effect alone could not explain the good dispersion of PCE-Cs on cement. This may be because the water-reducing and dispersing performance of PCE-Cs was more strongly affected by the steric hindrance effect of the side chains. In conclusion, the zeta potential test indirectly reflects the strength of electrostatic repulsion between cement particles by determining the charge on cement particles and then evaluates the dispersing ability of cement. However, the strength of the dispersibility of cement is not only determined by the zeta potential value but also needs to be combined with other microscopic properties to make a comprehensive judgment.

### 3.10. Adsorption Layer Thickness

**Figure 13:**
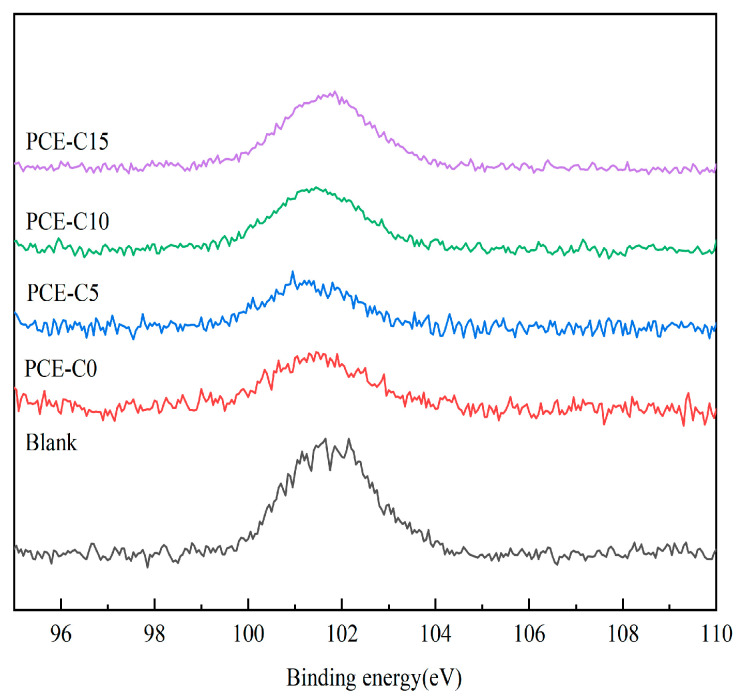
Si2p spectrum of cement particles after the addition of PCE-Cs.

The excellent dispersion performance of PCE on cement exhibited at a low dosage could not be explained by the DLVO electrostatic repulsion theory alone, so Yoshioka et al. [8] proposed the steric hindrance effect based on the colloidal stabilization system. PCE is adsorbed on the cement through the anionic groups, such as carboxyl groups on the main chain, and the long side chains of polyoxyethylene provide the steric hindrance effect to achieve the effect of dispersing cement. The long side chain can make the cement surface form a thick adsorption layer, which consists of water-reducing agents. The greater the thickness of the adsorption layer, the greater the steric hindrance provided by the PCE and, therefore, the greater the dispersing ability of cement. When the adsorption layer is present on the surface of the cement particles, it masks the Si and reduces its photoelectronic strength. At the same time, the Si element only exists in cement. It is possible to determine the degree of reduction in the photoelectron intensity of Si2p photoelectrons after passing through the water-reducing agent adsorbent layer by XPS and calculate the thickness of the adsorbent layer according to the calculation formula in the literature [40]. As shown in Figure 13, the greater the decrease in Si peaks after adsorption, the thicker the adsorption layer of the water reducer. The adsorption layer thicknesses of PCE-C0, PCE-C5, PCE-C10, and PCE-C15 on the surface of cement particles can be calculated as 1.80 nm, 2.17 nm, 1.63 nm, and 1.21 nm, respectively, according to the formulae, as shown in Table 9. The thicknesses of the adsorption layers of PCE-C0, PCE-C5, and PCE-C10 are larger, so they provide larger steric hindrance. PCE-C15 has the smallest adsorption layer thickness compared with other water-reducing agents, so the steric hindrance effect is weaker, and the effectiveness of dispersion to cement is not as good as other water-reducing agents.

**Figure 14:**
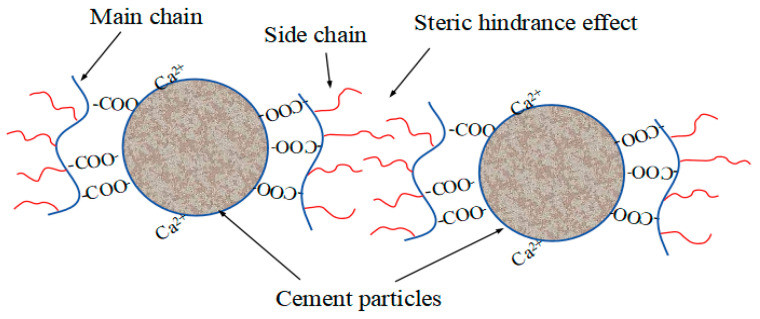
Dispersal mechanisms of PCE-Cs.

Based on the zeta potential test and adsorption test, the dispersion mechanism of PCE-Cs contains two main aspects. First, the carboxyl group on the main chain of PCE-Cs is negatively charged. The carboxyl group will form a double-layer structure with a positive charge on cement. Therefore, the surface of cement shows a negative charge. Electrostatic repulsion occurs between cements due to the same polarity charges, which in turn prevents the cement from forming a flocculent structure. From the results of the zeta potential test, it can also be found that PCE-Cs increase the absolute value of the zeta potential, so the repulsion between the cements becomes larger. On the other hand, as shown in Figure 14, the long side chains of PCE-Cs enter the liquid phase and form an adsorption layer. Therefore, a steric hindrance effect is generated between cements [47]. From the adsorption layer thickness test results, it was found that PCE-C5 has a larger adsorbent layer thickness and better dispersibility.

## 4. Conclusions

In this study, a method was proposed to synthesize a biomass polyether monomer, AHEC, via etherification and esterification modifications of natural cellulose. A new type of biomass concrete water-reducing agent was developed by partially replacing the traditional fossil-based raw material polyether monomer TPEG with AHEC as a key component. The novelty lies in the fact that the cellulose derivatives are endowed with highly efficient adsorption and dispersion ability through the modification routes of ethylene oxide etherification and acrylate esterification. So, they can effectively replace part of the traditional water-reducing agent main materials. The molecular structure of AHEC and traditional water-reducing agent components synergize to play the role of steric hindrance effects, which enhances the dispersion of cement. Experiments show that the water-reducing agent has good water-reducing dispersibility and fluidity retention. The water reduction in PCE-C5 can reach 27.3%, and there is no loss of 2 h flow of cement. While maintaining the same flowability of cement mortar, it can increase the mechanical strength by 30% to 40%. In addition, it also exhibits delaying properties. While improving water reduction and workability, it realizes the substitution of nonrenewable petroleum-based raw materials. This reduces carbon emissions and fossil resource consumption. And, because the raw material is derived from renewable biomass, the sustainability and environmental friendliness of the product are enhanced.

This study aimed to enhance the comprehensive utilization of cellulose and other biomass materials, reduce the waste of resources, and promote the transformation of renewable resources into a value-added direction. On this basis, we explored the use of biomass materials as raw materials to develop environmentally friendly, high-performance concrete water-reducing agents through chemical or physical modification and other means. This research pathway can help broaden the application areas of biomass resources. In addition, it is expected to effectively promote the development of the concrete admixture industry in a greener and more diversified direction.

This study lays a good foundation for the development of environmentally friendly biomass polycarboxylic acid water-reducing agents. Future research directions could involve the following aspects: The environmental impacts of PCE-Cs should first be assessed over their entire life cycle. The key indicators, such as carbon footprint and energy consumption, should be quantified and analyzed in comparison with traditional PCE. This will provide a solid scientific basis for the environmental friendliness of biomass water reducers. Secondly, the effect of PCE-Cs on the long-term durability of concrete, such as freeze–thaw resistance, carbonation resistance, and so on, should be studied in depth. The effect of PCE-Cs on the durability performance of concrete under simulated service environments should be investigated. This is essential to promote the application of PCE-Cs in practical engineering. Finally, advanced characterization techniques such as dynamic light scattering (DLS) and AFM are combined to further study the chain conformation of PCE-Cs in solution and reveal the relationship between microstructure and macroscopic properties. This will help to comprehensively assess the performance advantages and potential risks of water-reducing agents. And it can provide a solid design basis for optimizing performance.

## Figures and Tables

**Table 1:** Chemical composition and content of cement.

Component	LOI	Al_2_O_3_	CaO	MgO	Fe_2_O_3_	SO_2_	SO_3_
Content(%) *	2.98	5.85	70.45	1.31	3.03	18.99	2.79

* Cement is produced by Nanjing Longtan Town Cement Factory (Nanjing, China); The data comes from producers’ technical inspection reports.

**Table 2:** Technical metrics.

Specific Surface Area (m^3^/Kg)	Setting Time (min)		Compressive Strength (MPa)		Flexural Strength (MPa)	
	Initial	Final	3d	28d	3d	28d
367	315	380	23.6	45.1	4.6	7.4

**Table 3:** Technical indicators of coarse aggregates.

Aggregate Size	Proportion (%)	Percentage Passing (%)							
		31.5	26.5	19	16	13.2	9.5	4.75	2.36
20~30 mm	45	98	67.4	3.9	0.8	-	-	-	-
10~20 mm	55	100	100	96.9	75.0	6.9	5.3	0.6	-

**Table 4:** Technical indicators of fine aggregates.

Aggregate Size	Fineness Modulus	Percentage Passing (%)					
		4.75	2.36	1.18	0.60	0.30	0.15
medium sand	2.7	4	17	31	54	84	96

**Table 5:** Chemicals used in the paper.

Chemicals	Abbreviations
acrylic acid	AA
Hydroxyethyl acrylate	HEA
Isopentenyl polyoxyethylene ether	TPEG
Hydroxyethyl cellulose	HEC
ethylene oxide	EO
cyclohexane	CYH
P-toluenesulfonic acid	PTS
L-Ascorbic acid	Vc
mercaptopropionic acid	MPA
acryloyl hydroxyethyl cellulose	AHEC
Polycarboxylate superplasticizer	PCE
Biomass Polycarboxylate superplasticizer	PCE-Cs
calcium hydroxide	CH
Calcium Silicate Hydrate	C-S-H
Aluminate Ferrite tri-sulfate	AFt

**Table 6:** Mass of raw materials needed to synthesize PCE-Cs.

PCE-Cs	TPEG/g	AHEC/g	AA/g	HEA/g	H_2_O_2_/g	Vc/g	MPA/g
PCE-C0	166	/	10	24.25	0.82	0.275	0.81
PCE-C5	157.7	8.3	10	24.25	0.82	0.275	0.81
PCE-C10	149.4	16.6	10	24.25	0.82	0.275	0.81
PCE-C15	141.1	24.9	10	24.25	0.82	0.275	0.81

**Table 7:** Strength of cement mortar.

PCE-Cs	Compressive Strength (MPa)			Flexural Strength (MPa)		
	3d	7d	28d	3d	7d	28d
Blank	23.6	35.2	45.1	4.6	5.7	7.4
PCE-C0	34.7	46.3	58.0	6.1	7.4	8.7
PCE-C5	35.2	47.8	59.8	6.5	7.6	9.1
PCE-C10	33.8	47.0	56.5	5.8	7.0	8.4
PCE-C15	32.6	44.7	53.4	5.6	6.9	8.1

**Table 8:** Gel permeation chromatography of PCE-C0 and PCE-C5.

PCE-Cs	Mw (g/mol)	Mn (g/mol)	PDI	Polyether Monomer Conversion (%)
PCE-C0	57,109	30,331	1.88	91.7
PCE-C5	73,404	38,618	1.90	89.3

**Table 9:** The thickness of adsorption layer.

	PCE-C0	PCE-C5	PCE-C10	PCE-C15
*hv*/eV	1486.60	1486.60	1486.60	1486.60
*E*_b_/eV	101.45	101.30	101.50	101.60
*E*_k_/eV	1385.15	1385.30	1385.10	1385.0
*I*_0_	2012.41	2012.41	2012.41	2012.41
*I*	1296.52	1185.65	1351.54	1495.85
*b*/nm	1.80	2.17	1.63	1.21

## Data Availability

The original contributions presented in this study are included in the article. Further inquiries can be directed to the corresponding author.
